# CAR T-Cell Immunotherapy in Neuroautoimmune Diseases: Focus on the Central Nervous System

**DOI:** 10.3390/biomedicines14020296

**Published:** 2026-01-29

**Authors:** Fotis Demetriou, Maria Anagnostouli

**Affiliations:** 1 Medical Degree English Program, National and Kapodistrian University of Athens—NKUA, 11528 Athens, Greece; fotisdemetriou2000@gmail.com; 2 Multiple Sclerosis and Demyelinating Diseases Unit, Center of Expertise for Rare Demyelinating and Autoimmune CNS Diseases, 1st Department of Neurology, School of Medicine, National and Kapodistrian University of Athens—NKUA, Aeginition University Hospital, 11527 Athens, Greece; 3 Research Immunogenetics Laboratory, 1st Department of Neurology, School of Medicine, National and Kapodistrian University of Athens—NKUA, Aeginition University Hospital, 11528 Athens, Greece

**Keywords:** CAR T-cells, CNS autoimmunity, B-cells, DMTs, MS, NMOSD, MOGAD, autoimmune encephalitis (AE), HLA

## Abstract

The treatment of central nervous system (CNS) autoimmune diseases has evolved from broad immunosuppression toward targeted disease-modifying therapies (DMTs). While current DMTs effectively control inflammatory activity in many patients, unmet needs remain, including persistent compartmentalised CNS pathology, limited tissue penetration, and the cumulative burden of chronic therapy. Chimeric antigen receptor (CAR) T-cell therapy represents a novel “living” immunotherapy capable of antigen-specific cellular depletion. Although currently approved only for B-cell malignancies, CAR T-cells are increasingly being explored in CNS autoimmunity leveraging their capacity for autonomous cytotoxicity and expected access to immune cells within protected CNS niches following a potentially single intervention. In this review, we examine CAR T-cells in the context of CNS-autoimmunity, we outline principles derived from oncologic applications, assess current DMTs, their limitations and side effects, and define parameters where CAR T-cells may offer added value. We discuss biological and practical requirements for broader clinical application, as currently they are investigated only for the very severe and refractory cases where all alternative treatments have failed. We further review the plasticity of CAR constructs, distinguishing clinically advanced platforms from early proof-of-concept approaches. Finally, we summarise clinical experience from 15 patients with CNS autoimmunity treated with CAR T-cells and review ongoing or planned trials that include such patients. We conclude that CAR T-cell therapy remains investigational for severe, treatment-refractory disease, with future applicability dependent on demonstrable efficacy, safety, cost, and feasibility beyond existing DMTs.

## 1. Introduction

Neuroautoimmune conditions of the CNS including Multiple Sclerosis (MS), Neuromyelitis Optica Spectrum Disorders (NMOSD), Myelin Oligodendrocyte Glycoprotein Antibody-Associated Disease (MOGAD), and Autoimmune Encephalitis/Encephalomyelitis (AE), are characterised by demyelination and/or axonal degeneration, resulting from the activation of inflammatory responses against CNS antigens [1]. Historically, potent corticosteroids and other systemic immunosuppressants were used. This approach exposed patients to increased toxicity and an elevated risk for infections and development of malignancies, due to the chronic and extensive immune compromise. The development of DMTs shifted this paradigm by enabling for a more selective and efficacious targeting of immune mediators, without broad immunosuppression. Such DMTs were introduced for MS with IFN-beta, and Glatiramer Acetate in the 1990s [2,3], and monoclonal antibodies (mAbs), later, in the 2000s along with other, mainly oral pharmaceutical agents, targeting primarily the neuroinflammatory component of the disease. MAb-based therapies resulted to be very efficacious in MS and other CNS autoimmune diseases by targeting various mediators of autoimmunity which include pro-inflammatory cytokines (e.g., Tocilizumab, anti-IL6); (2) integrins which facilitate lymphocyte entry into the CNS via the blood–brain barrier (BBB) (Natalizumab); (3) immune cell populations, primarily B-cells (e.g., Rituximab, anti-CD20); and (4) Complement proteins (e.g., complement’s C5 with Ravulizumab) (see Table 1).

Such immunotherapeutic agents have now been used for decades in adult patient cohorts and are also promising targeted immunotherapies for paediatric populations [4,5]. Unmet needs remain though regarding their immunological frame of cellular targeting, with the most profound one being the elimination of CNS-located mediators of inflammation. For example, mAbs being large and charged polypeptides, are greatly intercepted by the BBB, resulting in CNS concentrations which are negligible compared to the relative in the blood circulation. A study comparing rituximab’s levels in the cerebrospinal fluid (CSF) with the peripheral blood circulation, showed that the CSF had only 1% of the corresponding serum levels [6]. Another preclinical study quantifying ocrelizumab in mouse brain, reported that only 0.1–1% of circulating monoclonal antibodies entered the CNS [7]. This biodistribution profile is expected to be a common characteristic of all therapeutic agents which involve large and/or charged molecules.

Their inability to penetrate the BBB restricts their effects almost exclusively to peripheral immune mediators and leave compartmentalised CNS-resident immune populations intact. More recently developed small-molecule therapies including Bruton’s tyrosine kinase inhibitors (BTKIs), achieve measurable CNS biodistribution and aim to deplete CNS-resident immune mediators [8]. Similarly, CAR T-cells are also investigated in their capacity to exhibit significant CNS biodistribution and eliminate currently inaccessible CNS-resident immune cells, this is a key justification for their investigation in CNS autoimmune diseases.

**Figure 1:**
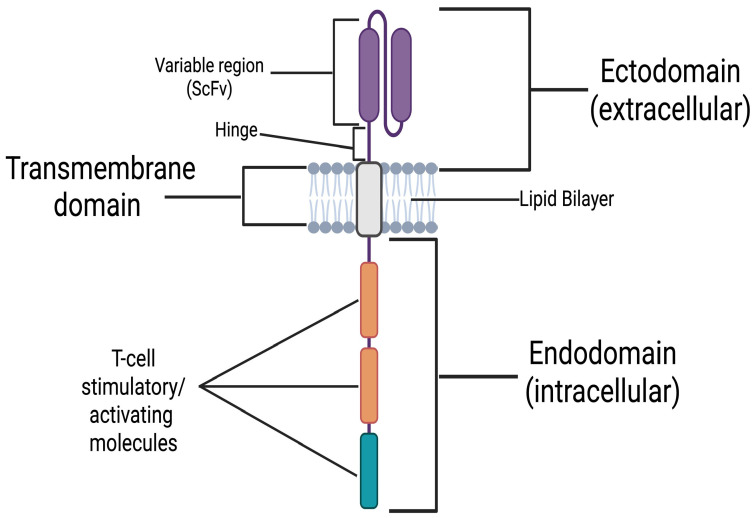
Schematic representation of the chimeric antigen receptor (CAR) consisting of 3 main regions: (1) the ectodomain (extracellular), the antigen-binding domain made from a single-chain variable fragment (scFv) derived from an antibody with affinity and specificity for that antigen, connected to a hinge region that provides flexibility; (2) the transmembrane domain, which anchors the receptor in the T-cell membrane; and (3) the endodomain (intracellular), which contains stimulatory/activating molecules. The endodomains of different CARs share CD3ζ derived from the T-cell receptor (TCR) (in blue), and one or two additional co-stimulatory molecules (CMs) commonly from CD28 and 4-1BB (in orange) [16,17,18]. “Created in BioRender. Demetriou, F. (2025) https://BioRender.com/jw8pks9” (accessed on 14 December 2025).

The CAR is an engineered transmembrane protein receptor, formed by linking an antigen-recognition domain (extracellular) to T-cell activating signalling motifs (intracellular) (see Figure 1). CAR T-cells are human T-cells, induced to express these receptors to create a “living drug”. CARs bind a specific cellular antigen and activate the CAR T-cells’ cytotoxic mechanisms to eliminate the cell that expresses it.

**Figure 2:**
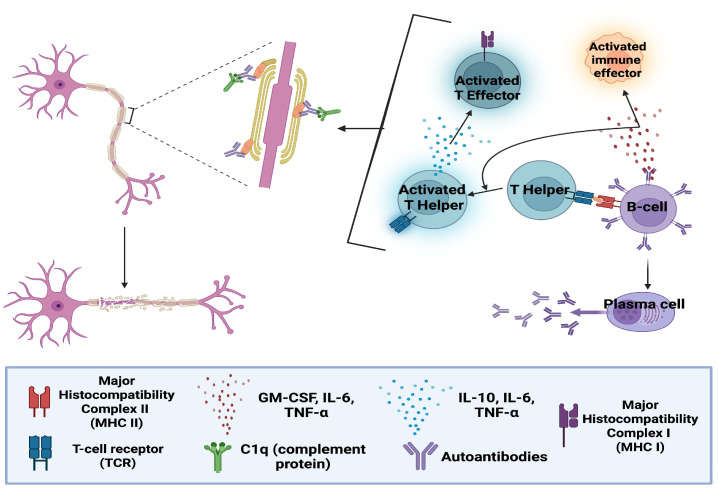
B-cell contribution in neurodemyelination/neurodegeneration. Activated B-cells present central nervous system (CNS) antigens and activate CD4 (+ve) T-helper cells, via major histocompatibility complex II (MHC II)/T-cell receptor (TCR) interaction. Activated CD4 (+ve) T-cells in-turn activate CD8 (+ve) T-effectors against these CNS antigens. B-cells also differentiate into plasma cells which secrete autoantibodies against CNS antigens. Antibody binding opsonises or “tags” the cell for destruction from the complement system (via C1q recognition of bound antibody) and/or other immune effectors. B-cell secretion of pro-inflammatory cytokines also recruits and activates additional immune effectors [12] “Created in BioRender. Demetriou, F. (2025) https://BioRender.com/jw8pks9”. (Accessed date on 14 December 2025).

All currently approved CAR T-cell therapies are used in B-cell malignancies, targeting either CD19 or B-cell maturation antigen (BCMA) (+ve) B-cells. Long-term follow-up data demonstrate an extensive and durable B-cell-depleting capacity [9]. B-cells also have a consistent and substantial contribution to the pathophysiology of all major CNS autoimmune conditions. Their involvement includes promotion of inflammation with cytokine and chemokine secretion, autoantibody production and antigen presentation with subsequent activation of autoreactive T-cells and other effectors (See Figure 2). As a result, mAb-based DMTs targeting B-cells, remain central for disease management [10,11,12,13]. Observations from CAR T-cell use in oncology overall include a profound B-cell depletion, deep tissue penetration, and the flexibility to target different cell types/subtypes. These characteristics, collectively support CAR T-cell investigation in CNS autoimmune conditions. Sixteen clinical trials currently active or planned which are discussed in Section 7, include CNS autoimmunity patients who have failed to respond to alternative treatments, and present with a severe disease phenotype, with uncontrolled progression.

Drawbacks of existing DMTs such as mAb-based therapies, include among others the requirement for repeated dosing to achieve and maintain disease remission due to their finite half-life and the eventual reconstitution of the targeted immune populations. This treatment pattern results in chronic and cumulative associated side effects, as all CNS autoimmune conditions are chronic.

Treatments that aim to achieve prolonged disease remission with fewer interventions include the so-called immune reconstitution therapies (IRTs) [14]. In the context of anti-B-cell therapies, IRTs, compared to mAbs, aim to induce a significantly deeper elimination of B-cells with each dosing. This more extensive elimination of the B-cell pool results in regeneration of a new B-cell repertoire predominantly from hematopoietic progenitor stem cells rather than from mature dividing B-cells. The greater number of pathogenic cells eliminated can reduce the number of pathogenic daughter-cells generated during immune reconstitution, potentially resulting in a longer-lasting “healthy” immune repertoire and prolonged disease remission. While this more extensive depletion achieved by IRTs may reduce treatment frequency, the greater potency of the treatment might result in more severe adverse events immediately after their administration.

Compared to IRTs, CAR T-cell therapies have a distinct advantage. As a “living therapy”, they can potentially persist and continue to express the CAR, enabling an even more prolonged elimination of their target. This can be considered both a beneficial and a potentially dangerous characteristic: beneficial in that it may function as a one-time treatment, and dangerous in that its effects may be unpredictable. The persistence and activity of CAR T-cells cannot be predicted with certainty, as they are influenced by multiple parameters [15]. Unlike conventional drugs, which are defined by a finite half-life, these “living therapies” are influenced by factors including the fitness of the T-cells collected for CAR T-cell manufacturing and the cytokine profile of the patients receiving them.

CAR T-cell therapies are also associated with acute and delayed toxicities, high manufacturing costs, and logistical challenges, which currently limit their use in aggressive and/or relapsing cases where all alternatives have failed. Continued development of safety-mitigation strategies and cost-reduction approaches will be essential to establish CAR T-cell therapies as a clinically realistic option for a broader range of patients with CNS autoimmunity.

## 2. The CAR and Its Evolution, “Hijacking” Cytotoxic T-Cell Capacities to Eliminate Cellular Targets

### 2.1. The Chimeric Antigen Receptor (CAR)

**Figure 3:**
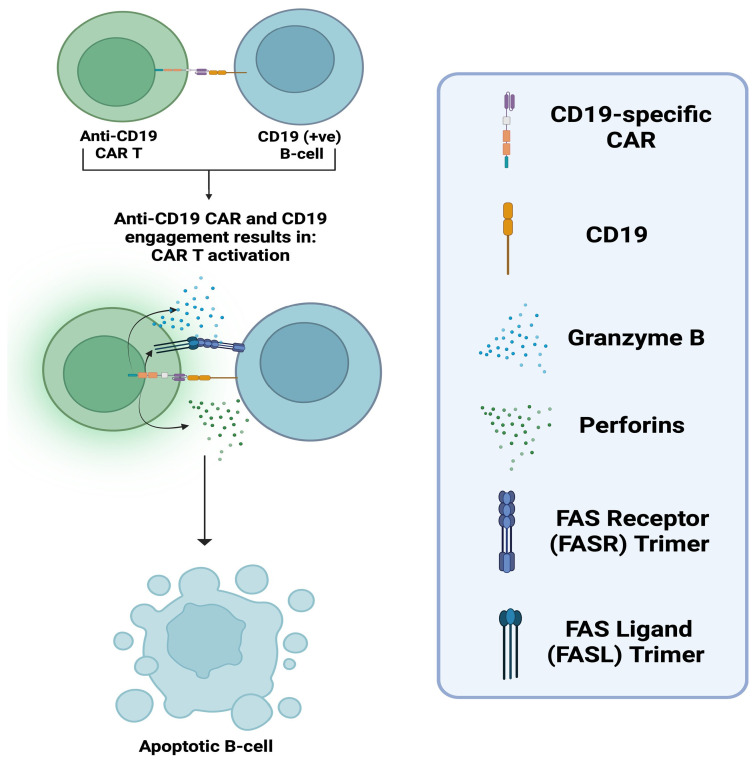
Schematic illustration of the mechanism by which a CAR T-cell e.g., anti-CD19, recognises and eliminates CD19 (+ve) B-cells. Binding of anti-CD19 CAR T-cell to CD19 on the targeted B-cell, triggers CAR T-cell activation. Activation results to the release of perforins and granzyme B. Perforins form pores on the B-cell-membrane, facilitating Granzyme B entry into the cell, which in-turn activates intrinsic apoptotic pathways. Engagement of the FASL on the CAR T-cell with FASR on the B-cell activates apoptotic pathways via the receptor-mediated extrinsic pathway [20]. “Created in BioRender. Demetriou, F. (2025) https://BioRender.com/jw8pks9” (accessed on 14 December 2025).

The CAR can be divided into three major domains [16]: the (1) ectodomain, the (2) transmembrane domain, and the (3) endodomain (See Figure 1). The ectodomain is the extracellular portion of the receptor. It includes a variable-binding region with affinity and specificity for an antigen expressed on a cellular target. The variable-binding region is a single-chain variable fragment (scFv) derived from the heavy and light chains of an antibody with affinity and specificity for the target antigen. The scFv is connected to a hinge region, which bridges it to the transmembrane domain. The hinge increases binding capacity at the immunological synapse by providing flexibility. This flexibility allows the binding region to adopt different spatial orientations to facilitate a more efficient scFv-antigen interaction [17]. Next comes the transmembrane domain, which, as the name implies, spans the lipid bilayer and anchors the receptor in place. Finally, the endodomain, located intracellularly, translates extracellular antigen recognition into signalling cascades, activating the CAR T-cell, resulting in the elimination of the cellular target [18]. The activated CAR T-cell eliminates its target by the secretion of perforins and Granzyme B, and the upregulation of its Fas ligand (FASL). As it is well known, perforins open pores on the target’s membrane to facilitate Granzyme B entry in the target’s cytoplasm which in-turn activates intrinsic apoptotic pathways. FASL binds FAS receptor (FASR) on the target which activates its extrinsic apoptotic pathways (See Figure 3). The risk of perforin/granzyme and FASL-mediated apoptosis in inducing bystander damage to neighbouring neural cells, must also be addressed regarding the safety profile of this novel therapy in the context of CNS autoimmunity.

Physiologically, the activation of T-cells requires the T-cell receptor (TCR) to recognise non-self antigens presented by self-major-histocompatibility-complex (MHC) molecules on the target’s cell surface, and to receive co-stimulatory signals from secondary receptors for a successful T-cell activation. A breakthrough introduced by CAR technology is that its intracellular signalling motifs can exert sufficient activatory-signalling independently of co-stimulation, while its ectodomain can recognise antigens without the need for them to be processed and presented through MHC molecules. This innovation renders CAR T-cell activation independent of both MHC- and co-stimulatory signals, effectively allowing us to “hijack” the major physiological checkpoints that typically regulate a T-cell-mediated cytotoxic response. However, this characteristic has also led to them being described as “biological serial killers” [19] owing to their potentially aggressive behaviour.

### 2.2. The History and the Different Generations of CARs

**Figure 4:**
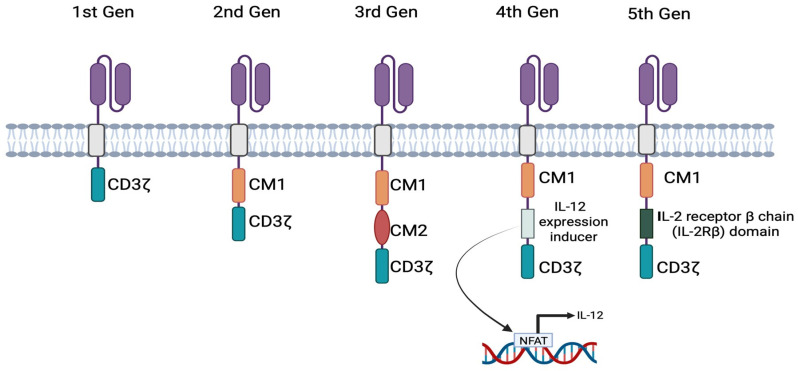
Schematic of the five generations of chimeric antigen receptors (CARs), varying in their intracellular/signalling domain. The 1st generation includes only the CD3ζ signalling domain (homologous to the native T-cell receptor); the 2nd generation adds a single co-stimulatory motif (CM1); the 3rd generation incorporates two co-stimulatory motifs (CM1 and CM2); the 4th generation known as T-cells Redirected for Universal Cytokine-mediated Killing (TRUCKs) introduces an IL-12-expressing CM that activates Nuclear Factor of Activated T-cells (NFAT) which as the arrow shows translocates in the nucleus, binds its promoter and induces the expression of IL-12. The 5th generation includes an IL-2 receptor β-chain (IL-2Rβ) domain to activate the JAK-STAT pathway to further promote activation and survival [22,23,24,25,26,27,28,29,30]. “Created in BioRender. Demetriou, F. (2025) https://BioRender.com/jw8pks9” (accessed on 14 December 2025).

CAR T-cell therapy evolved from early adoptive cell therapies, notably the work by Rosenberg et al. in 1988 [21] demonstrating tumour regression in metastatic melanoma using ex vivo expanded tumour infiltrating lymphocytes, which paved the way for genetically engineered T-cells. CARs are synthetic receptors that redirect T-cells toward predefined cell surface antigens in an MHC independent manner; clinically approved CARs predominantly target cluster of differentiation (CD) antigens in B-cell malignancies, most notably CD19 or BCMA. All CAR generations share the CD3ζ signalling domain derived from the native T-cell receptor, while differing in their inclusion of co-stimulatory molecules (CMs) that modulate activation strength and persistence. First generation CARs, introduced by Eshhar et al. in 1993 [22], fused an scFv to CD3ζ alone and showed limited efficacy due to insufficient T-cell activation. Second generation CARs added a single CM such as CD28 or 4-1BB [23,24,25], markedly improving proliferation, cytokine secretion and in vivo persistence; importantly, all CAR T-cell therapies currently approved for clinical use belong to this second-generation design. Regarding CM selection, CD28 is considered to drive a rapid, potent effector responses, whereas 4-1BB to promote a slower expansion with enhanced persistence and memory formation [24]; additionally, CD28-based constructs are more prone to tonic signalling, an antigen-independent CAR activation that can drive premature T-cell exhaustion [24], a consideration of particular relevance in autoimmune applications where a potentially prolonged, controlled activity is desired over acute cytotoxicity. How these distinct functional profiles translate to therapeutic outcomes in CNS autoimmunity remains an important parameter for future investigation. Third generation CARs incorporate multiple CMs [26] to further enhance expansion, though at the cost of increased risk of T-cell exhaustion [27]. Fourth generation CARs, or TRUCKs, include an NFAT responsive promoter that drives inducible IL-12 expression upon antigen engagement to remodel the tumour microenvironment [28,29]. Fifth generation CARs further integrate the IL-2 receptor β chain to activate JAK–STAT signalling, promoting sustained proliferation and prolonged functional activity of CAR T-cells [30] (See Figure 4).

## 3. Current Disease-Modifying Therapies in CNS Autoimmunity: Capabilities, Limitations, Unmet Needs, and the Clinical Reality of CAR T-Cell Applications

### 3.1. DMTs and CAR T-Cells in CNS Autoimmunity

**Figure 5:**
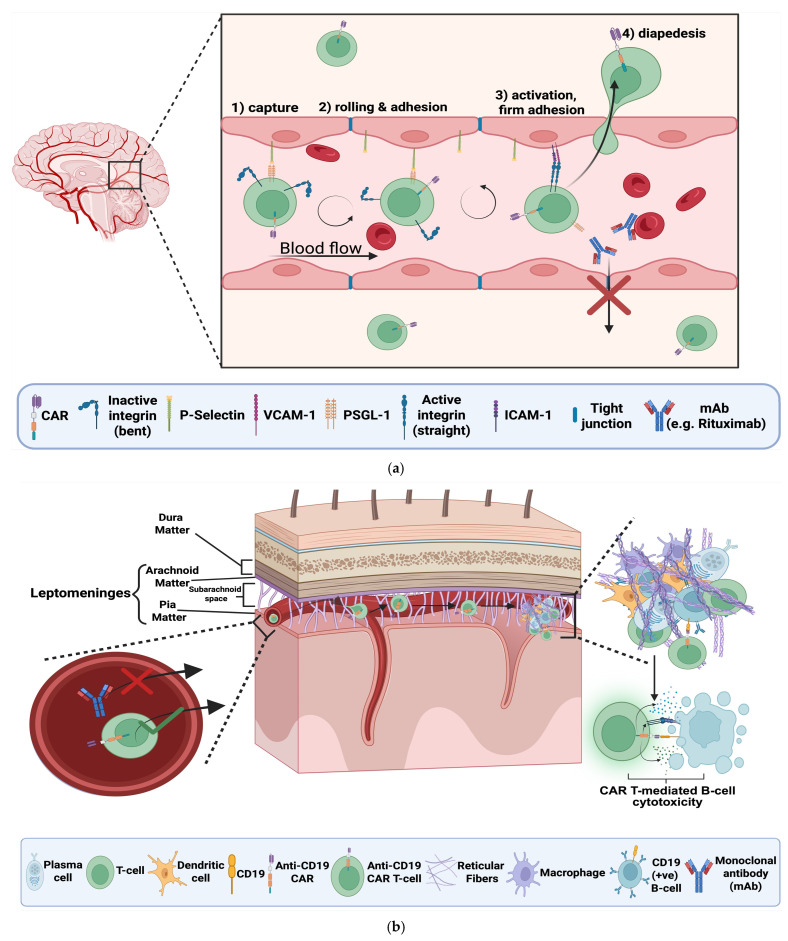
(**a**) Illustration of the differential ability of chimeric antigen receptor (CAR) T-cells versus monoclonal antibodies (mAbs) to cross the blood-brain-barrier (BBB). The expanded inset depicts the leukocyte adhesion cascade enabling CAR T-cell diapedesis across the BBB endothelium. The process involves four steps: (1) capture, where P-selectin on endothelium binds P-selectin glycoprotein ligand-1 (PSGL-1) on activated CAR T-cells; (2) rolling and adhesion, as CAR T-cells slowly roll with the flow of blood, depicted with the circular arrows, until their integrins engage with endothelial vascular cell adhesion molecule-1 (VCAM-1) and intercellular adhesion molecule-1 (ICAM-1) on the endothelium; (3) activation and firm adhesion, mediated by integrin conformational change from inactive (bent) to active (straight); and (4) diapedesis into the CNS via disassembled endothelial tight junctions. In contrast, mAbs (e.g., Rituximab) are shown unable to pass the BBB, due to the existence of dense/assembled tight junctions between endothelial cells [36,40,48]. “Created in BioRender. Demetriou, F. (2025) https://BioRender.com/jw8pks9” (accessed on 14 December 2025) (**b**) Proposed elimination mechanism of B-cells e.g., CD19 (+ve) within tertiary lymphoid follicles (TLFs) in the subarachnoid space of the leptomeninges of multiple sclerosis (MS) patients with chimeric antigen receptor (CAR) T-cells. CAR T-cells successfully migrate into the leptomeningeal compartment (Bruton’s tyrosine kinase inhibitors (BTKIs) are also theoretically partially able to do so), while monoclonal antibodies (mAbs) do not. The left inset depicts this differential ability. Once CAR T-cells enter the CNS, they travel through its compartments, e.g., the subarachnoid space, to potentially reach TLFs as depicted with the arrows. The inset on the right shows the pathogenic cellular organization within TLFs, consisting of among others: B-cells, plasma cells, macrophages, dendritic cells, and reticular fibres. The lower panel depicts CAR T-cell–mediated cytotoxicity directed against CD19 (+ve) B-cells [36,40,47,48,60]. “Created in BioRender. Demetriou, F. (2025) https://BioRender.com/jw8pks9” (accessed on 14 December 2025).

DMTs available or under investigation for the treatment of CNS autoimmunity are summarised in Table 1. Current DMTs achieve substantial control of relapsing inflammatory activity through diverse immunomodulatory, immunodepletive, and immune-reconstituting mechanisms [14,21,22,23,24,25,26,27,28,29,30,31,32,33]. Injectable immunomodulators and oral agents show favourable and predictable pharmacokinetic/pharmacodynamic (PK/PD) and safety profiles, but the requirement of repeated dosing results in a prolonged and cumulative burden of their associated side-effects [34,35]. Moreover, especially mAb-based DMTs show limited efficacy in highly active disease that is driven by compartmentalised CNS immune cells [36] (See Figure 5a) which remains a major unmet need in the treatment of a subset of CNS autoimmunity patients.

Emerging small-molecule therapies, such as BTKIs have shown promise in potentially addressing biodistribution-related limitations with measurable CNS concentrations [37,38,39]; nevertheless, their long-term impact on CNS-resident immune compartments and inflammation remains under active investigation. In comparison with the potentially prolonged cytotoxic activity of CAR T-cells, BTKIs act by modulating the activity of their target by competing for the ATP-binding site of BTK, blocking downstream signalling cascades essential for B-cell activation, proliferation, and survival, while also modulating microglial function through inhibition of Fc receptor and toll-like receptor signalling pathways within a finite time-frame [37].

Unlike conventional drugs including BTKIs, which rely on passive diffusion for their trafficking to tissues, CAR T-cells are in theory able to utilise active, lymphocyte-trafficking processes also known as the “Leukocyte Adhesion Cascade”. Activated lymphocytes express adhesion molecules that engage binding partners upregulated on inflamed endothelium, after chemokine gradients guide them to these sites. Interaction of these molecules eventually leads to their arrest and diapedesis into adjacent tissue [40]. Interestingly, natalizumab, a current DMT, binds lymphocyte integrins to prevent their interaction with their BBB partners, aiming to reduce the number of pathogenic lymphocytes infiltrating the CNS as a mean to downregulate autoimmune responses [41]. In contrast, in the context of CAR T-cells, these same cascades if employed, are considered of great benefit, since trafficking of CAR T-cells to the CNS is a desirable event. This theoretical trafficking capacity, if successful, raises hopes for the subsequent engagement and elimination of CNS-resident targets, including those within TLFs (see Figure 5a,b).

CAR T-cells though, currently stand as a clinically realistic investigational option only for very severe and refractory cases of CNS autoimmunity, owing to high cost, complex logistics, significant adverse effects, and most importantly, the lack of substantial clinical data from their application in CNS autoimmunity.

### 3.2. Requirements for CAR T-Cells to Become a Clinically Real Treatment Option in a Broader Range of CNS Autoimmunity Patients

As outlined above, given the high cost, complexity, and risk profile of CAR T-cell therapies, their application/investigation is justified in patients with severe refractory disease, where alternative DMTs are ineffective potentially driven by CNS-resident lymphocytes or TLFs. This population is likely to represent a small but meaningful subset, rather than the broader relapsing MS, NMOSD and MOGAD populations that are currently managed to a great extend with the existing DMTs.

Second, safety must be demonstrated via ongoing clinical trials. While CRS and ICANS are in part expected to be milder compared to oncology due to the lower antigen burden, characteristics specific to CNS-autoimmunity including variable BBB integrity and alterted cytokine/chemokine profiles, can potentially result in unpredicable manifestations of these syndromes [42,43,44]. Risks associated with unpredictable PK/PD profiles, persistence, and in vivo activity may be mitigated by the incorporation of safety switches currently under investigation in preclinical models (not expected to be a clinical reality in the near future), or by the development of transient CAR T-cell therapies, such as mRNA-induced CAR T-cells, which have ongoing human clinical trials in peripheral neuroautoimmunity [45]. Exploring CAR T-cells in non-life-threatening conditions and especially in CNS autoimmunity patients that respond to current DMTs or do not present with very severe phenotypes of such conditions, would be ethically wrong, owing to the known severe adverse side-effects and the c urrently limited knowledge of their risk-benefit balance.

Third, CAR T-cell therapies must demonstrate durable clinical benefit that exceeds what is achievable with IRTs such as cladribine and alemtuzumab (mainly). While these agents already allow long treatment-free intervals, their effects on CNS-resident immune cells are indirect and incomplete [14]. CAR T-cells must therefore demonstrate superior durability, deeper depletion of pathogenic immune compartments, or direct targeting of CNS-resident lymphocytes to justify their use as a single-intervention strategy.

Finally, feasibility and cost considerations remain critical. As shown in Table 2, CAR T-cell therapies currently occupy the extreme upper end of treatment cost, exceeding even high-cost monoclonal antibody therapies such as complement inhibitors. For CAR T-cells to become clinically applicable beyond experimental or highly selected indications, advances in manufacturing scalability will be required. These may include “off-the-shelf” allogeneic CAR T-cell products or mRNA-induced CAR T-cells. Without such developments, CAR T-cell therapy is likely to remain confined to highly specialised centres and exceptional clinical scenarios.

### 3.3. TLFs and the Inability of Anti-B-Cell mAbs to Target Them Effectively

Antibodies are large, charged macromolecules whose passive diffusion across the BBB is severely restricted (See Figure 5a,b). A study analysing rituximab biodistribution demonstrated that CSF concentrations were below 1% of corresponding serum levels [6]. Consistent with this, a preclinical study quantifying ocrelizumab in mouse brain reported that only 0.1–1% of circulating mAbs enter the CNS from the systemic circulation [7].

Anti-B-cell mAbs, even with their limited CNS biodistribution, became a revolutionary DMT by introducing the capacity to selectively deplete immune-cells by targeting specific surface markers such as CD19 or CD20 on B-cells. mAbs cannot autonomously kill the cells they bind but require the aid of other immune effectors. When mAbs bind their cellular antigen, they opsonise “tag” the cell for elimination. Opsonised cells are eliminated via three mechanisms [46]: (1) antibody-dependent cellular cytotoxicity (ADCC), (2) antibody-dependent cellular phagocytosis (ADCP) and (3) complement-dependent cytotoxicity (CDC). ADCC involves natural killer (NK) cells, neutrophils (Neu), and/or macrophages (MΦ) that secrete cytotoxic granules to eliminate their targets. ADCP is largely mediated by MΦs, which phagocytose their targets. CDC eliminates opsonised cells when circulating complement proteins recognise a bound antibody and trigger cascades that recruit additional complement proteins to form a membrane-attack-complex (MAC), which forms a hole and kills the opsonised cell. In contrast, CAR T-cells are entirely self-sufficient. Furthermore, the accessory immune effectors required for mAb-mediated cytotoxicity previously discussed, must also access the CNS to exert their functions, constituting their biodistribution an additional limiting factor for successful target depletion. 

The presence of cerebrospinal fluid (CSF)–restricted oligoclonal bands (OCBs), reflecting intrathecal immunoglobulin synthesis, represents one of the most reliable diagnostic features distinguishing MS from NMOSD, AE and MOGAD. In MS, OCBs arise from peripherally activated B-cells that enter the CNS and undergo local differentiation, giving rise to antibody-secreting plasma cells and memory B-cells that may persist within TLFs, thereby sustaining intrathecal antibody production without the requirement of the involvement from their peripheral counterparts [47].

Particularly in secondary progressive MS (SPMS), meningeal TLFs are frequently observed and correlate with a more severe disease phenotype [47]. B-cells residing within these follicles are particularly resistant to mAb-mediated depletion. In addition to the minimal CNS biodistribution of mAbs, the follicular microenvironment provides survival signals and acts as a physical barrier, further hindering antibody penetration and effector-cell access. CAR T-cells are being explored, and wether they manage to overcome these access-related limitations remains speculative. The previously discussed mechanisms in Section 3.1, along with experience from CAR T-cell uses in CNS malignancies which show significant CAR T-cell CNS penetration [48], give promise for a similar scenario in their application in CNS autoimmunity.

Emerging BTKIs, by penetrating the CNS and dampening inflammation in meningeal TLFs, have shown promise in slowing neurodegeneration [38]. In the same way, if CAR T-cells manage to access and eliminate pathogenic B-cell and myeloid populations within these follicles, they may also theoretically reduce the local inflammatory drivers of neurodegeneration.

**Table 1:** Current DMTs and CAR T-cell in CNS-autoimmunity.

Class/Examples	Mechanism of Action	Patients	Efficacy	Cost	CNS Biodistribution	Side-Effects and Limitations	References
Injectable immunomodulators including Interferon-β, Glatiramer acetate	T-cell activity modulation	RRMS	Low-moderate	Low-moderate	Minimal	Injection reactions; limited efficacy in highly active disease	[31,36,49]
Oral immunomodulators e.g., Dimethyl fumarate, Teriflunomide	T-cell apoptosis, Anti-inflammatory	RRMS	Moderate	Moderate	Minimal	GI effects, repeated Lymphopenias and Hypogammaglobulinemia with each treatment	[32,35,36]
S1P receptor modulators e.g., Fingolimod, Siponimod, Ozanimod	Lymphocyte sequestration in lymph nodes	RRMS; SPMS	Moderate-High	Moderate-High	Partial (highest with Fingolimod)	Bradycardia, Infections	[36,50]
Anti-CD20 monoclonal antibodies e.g., Ocrelizumab, Ofatumumab, Rituximab (off-label)	B-cell depletion	RRMS; PPMS; NMOSD (off-label); MOGAD (off-label)	High mainly in RRMS	Ocrelizumab, Ofatumumab (High), Rituximab (low-moderate)	Minimal	Repeated and potentially prolonged Lymphopenias and Hypogammaglobulinemia with Immune Compromise	[33,36,51,52]
Immune reconstitution therapies (IRT) including Cladribine, Alemtuzumab	Extensive lymphocyte depletion with immune reconstitution	Highly active RMS by EMA; RMS by FDA	High to very high	High	Measurable CNS-distribution with Cladribine; Minimal with Alemtuzumab	Finite Lymphopenia with a transient Immune Compromise until immune reconstitution	[14,53]
Natalizumab	Integrin blockade	RMS	High	High	Minimal	PML	[41]
Tocilizumab	IL-6 Receptor blockade	NMOSD, Refractory MOGAD	High	Moderate	Minimal	UTIs, Upper respiratory infections, Anaemia, Hepatotoxicity	[54]
Ravulizumab	Complement blockade (anti-C5)	NMOSD	High to very high	Very High	Minimal	Meningococcal Infections	[55,56,57]
Mitoxantrone	Broad immunosuppression	Aggressive RRMS/SPMS (rarely used)	Moderate	Low	Detectable in CSF	Cardiotoxicity, Therapy-related leukaemia	[58]
BTK inhibitors e.g., Evobrutinib, Tolebrutinib	Interfere with B-cell and myeloid signalling	Phase II/III trials: RMS, RRMS, PPMS, SPMS	Potentially High	Potentially Moderate	Measurable in CSF with biological activity	Immunosuppression, Neutropenia, Lymphopenia, Anaemia, Thrombocytopenia	[8,38,39,59]
Anti-B-cell CAR T-cells e.g., anti-CD19	Antigen-specific elimination of cellular targets	Refractory Diseases where alternative treatments have failed (experimental)	Potentially Very High	Very High	Potentially high to very high	CRS, ICANS, Ppotentially prolonged Lymphopenias/Hypogammaglobulinemia, Unpredictable PK/PD profile and persistence	[9,40,48]

DMTs: Disease-Modifying Therapies; CAR T-cells: Chimeric Antigen Receptor T cells; CNS: Central Nervous System; RMS: Relapsing Multiple Sclerosis; RRMS: Relapsing-Remitting Multiple Sclerosis; PPMS: Primary Progressive Multiple Sclerosis; SPMS: Secondary Progressive Multiple Sclerosis; NMOSD: Neuromyelitis Optica Spectrum Disorder; MOGAD: Myelin Oligodendrocyte Glycoprotein Antibody–Associated Disease; EMA: European Medicines Agency; FDA: Food and Drug Administration; S1P: Sphingosine-1-Phosphate; C5: Complement Component 5; IRT: Immune Reconstitution Therapy; CSF: Cerebrospinal Fluid; PK/PD:Pharmacokinetic/Pharmacodynamic; GI: Gastrointestinal; UTIs: Urinary Tract Infections; CRS: Cytokine Release Syndrome; ICANS: Immune Effector Cell-Associated Neurotoxicity Syndrome; PML: Progressive Multifocal Leukoencephalopathy.

## 4. Immune Reconstitution in CNS Autoimmunity and What CAR T-Cells Might Have to Offer, Beyond Existing Therapeutic Means

**Figure 6:**
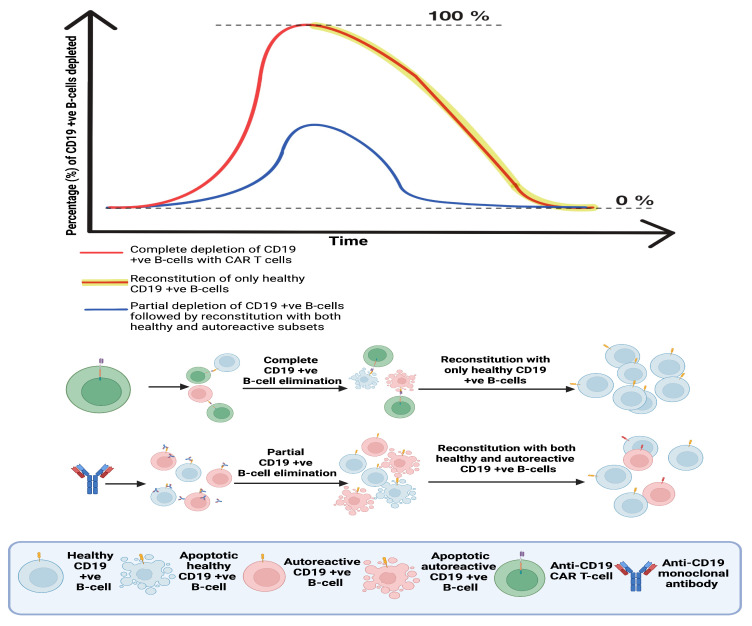
Proposed model of “immune resetting” specific populations of cells e.g., CD19 (+ve) B-cells with CAR T-cells. The graph shows that anti-CD19 CAR T-cells potentially induce rapid and complete depletion of CD19 (+ve) B-cells (healthy and autoreactive), followed by reconstitution with solely healthy CD19 (+ve) B-cells. In contrast, anti-CD19 monoclonal antibodies achieve only partial, transient depletion, permitting survival and subsequent re-emergence of both healthy and autoreactive CD19 (+ve) B-cells. “Created in BioRender. Demetriou, F. (2025) https://BioRender.com/jw8pks9” (accessed on 14 December 2025).

The concept of “resetting” the immune system in autoimmunity has garnered significant attention. The underlying rationale is that by deeply eliminating immune populations and allowing for their reconstitution, autoreactive subsets may be absent from the regenerated immune repertoire (See Figure 6). In principle, such an approach could enable durable disease remission with discontinuation of ongoing therapy.

Autologous hematopoietic stem cell transplantation (aHSCT), initially developed for hematologic malignancies, represents the most established immune resetting strategy in CNS autoimmunity and has been investigated in multiple sclerosis (MS) since the mid-1990s. While early protocols employed fully myeloablative conditioning with considerable toxicity prior to aHSCT, current regimens predominantly rely on lymphoablative rather than myeloablative chemotherapy, improving safety [61,62,63]. In MS, initial studies enrolling patients with advanced progressive disease showed limited benefit, whereas subsequent trials, including a phase II randomised controlled study demonstrating marked reductions in MRI disease activity and provided the rationale for larger studies [64]. Accumulating evidence now indicates that early use of aHSCT in patients with highly active relapsing–remitting MS (RRMS) results in profound suppression of clinical relapses and MRI activity, with disability improvement exceeding that achieved with DMTs [61]. Current consensus recommendations restrict aHSCT to young, ambulatory patients with short disease duration and ongoing inflammatory activity. Limited benefit observed in patients with advanced disability or progressive disease courses, may reflect the greater neuroplasticity in the young brain [65]. Despite the shift toward lymphoablative conditioning rather than myeloablative, aHSCT remains a non-selective intervention with acute and delayed side effects. Aute toxicities include pancytopenia, neutropenic fever and mucositis. Transient worsening of neurological symptoms is also common during and following aHSCT. Infertility is also common and recovery from it regresses with increasing age. Treatment related mortality has also been reported but has significantly decreased over the years dropping from 7.3% between 1995–2000 to 0.3% in post-2005 studies [63].

Beyond aHSCT, IRTs such as alemtuzumab and cladribine have emerged as less intensive approaches, that partially exploit the Immune-resetting paradigm [14]. These agents induce transient but profound lymphocyte depletion followed by gradual immune reconstitution, leading to sustained disease control in a subset of patients after limited treatment courses. In RRMS, both therapies have demonstrated durable suppression of disease activity beyond the period of drug exposure, supporting the concept that qualitative changes in the reconstituted immune repertoire may contribute to long-term efficacy. However, immune reconstitution following IRTs remains incomplete and variable, and these therapies are associated with distinct safety concerns, including secondary autoimmunity (particularly with alemtuzumab), infections, and prolonged lymphopenia.

In this context, CAR T-cell therapies offer a conceptual advantage over aHSCT and pharmacological IRTs by enabling antigen-defined immune depletion rather than global lymphoablation. As previously discussed in (Section 3.3), in addition to improved selectivity, CAR T-cells may theoretically achieve a more effective targeting of compartmentalised immune populations, including TLFs. Additionally, evidence supporting the immune-resetting potential of CAR T-cells has emerged from peripheral autoimmunity. Anti-CD19 CAR T-cell therapy induced profound and durable remissions in five patients with severe, multi-organ systemic lupus erythematosus (SLE) [66]. In this cohort, all immunosuppressive therapies were discontinued, drug-free remission was achieved in all patients, and B-cell populations reconstituted within 110 ± 32 days post-infusion, with no disease relapse during long-term follow-up. Notably, immune phenotyping demonstrated reconstitution dominated by CD21 (+ve)/CD27 (−ve) naïve and CD21 (+ve)/CD27 (+ve) memory B-cells, while pathogenic CD11c (+ve) and CD21^lo B-cell subsets commonly expanded in SLE were absent, suggesting qualitative immune reprogramming rather than simple depletion.

Despite these encouraging findings, their extrapolation to CNS autoimmunity must be approached with caution. All reported CAR T-cell cases in autoimmunity involved lymphodepleting chemotherapy prior to infusion, raising the possibility that observed clinical benefit may be partially or substantially attributable to conditioning regimens rather than CAR T-cell activity per se. Moreover, CAR T-cell therapies remain aggressive interventions thus while they represent a compelling extension of immune-resetting strategies and may offer theoretical advantages in targeting CNS-resident immune pathology, current evidence is insufficient to conclude that they can reliably induce an “immune reset” in CNS autoimmunity. Robust, controlled studies with long-term follow-up will be required to determine whether CAR T-cell therapies offer meaningful clinical advantages over established approaches in the context of immune reconstitution.

## 5. Adverse Side-Effects Associated with CAR T-Cell Therapies and a Way to “Switch Off” Their Activity as a Safety Measure

### 5.1. Adverse Side-Effects

Unfortunately, CAR T-cell therapy is associated with toxicities that can be life threatening in some patients. The most common severe toxicities are cytokine release syndrome (CRS) and immune effector cell–associated neurotoxicity syndrome (ICANS). Both are cytokine driven: CRS manifests as systemic inflammation with fever, hypotension, and hypoxia, whereas ICANS involves CNS inflammation presenting as encephalopathy with aphasia, tremor, and seizures. Key cytokines implicated include interferon γ, TNF α, and interleukins 1, 2, 6, 8, and 10. Grading for both syndromes relies on ASTCT criteria [67].

Clinically, ICANS usually emerges within days to a couple of weeks after infusion, and CRS also within several weeks after the infusion in almost 100% of patients that received anti-CD19 CAR T-cells. While mechanisms are not fully defined, ICANS is thought to arise from endothelial activation and BBB dysfunction, and CRS is the result of a cytokine “storm” from the extensive and rapid CAR T-cell activation [68].

Management of CRS and ICANs is syndrome specific. For CRS, IL-6 receptor blockade with Tocilizumab is standard, with corticosteroids added for severe or refractory cases. IL-1 blockade with Anakinra is increasingly used when CRS or ICANS is refractory to initial therapy. In contrast, isolated ICANS is managed primarily with corticosteroids as Tocilizumab is not reliably effective for ICANS in the absence of concurrent CRS [69]. 

In a report of 957 patients with hematologic malignancies who received Tisagenlecleucel or Axicabtagene Ciloleucel, both anti-CD19 CAR T-cell products, approximately 5% died from non-relapse causes within 30 days of their initial infusion [70].

Early experience with CD19-directed CAR T-cells in autoimmune diseases, including CNS autoimmunity, suggests lower rates and severity of CRS and ICANS than those observed in cancer. This is most logically due to a lower total antigen burden (B-cell populations) in autoimmunity compared to malignancies, resulting in milder CAR T responses and fewer pro-inflammatory killing byproducts released into the circulation [71]. In the largest reported cohort of 12 patients with treatment-refractory NMOSD treated with BCMA-directed CAR T-cells, all patients developed low-grade CRS, but notably, no patient developed ICANS [72]. However, all patients experienced grade ≥3 adverse events, predominantly hematologic toxicities related to conditioning chemotherapy, including leukopenia and neutropenia (100%), anemia (50%), and thrombocytopenia (25%), most of which resolved within four weeks. Infections occurred in 58% of patients, including cytomegalovirus reactivation, pneumonia, and coagulation disorders, underscoring the clinically relevant immunosuppressive burden of CAR T-cell therapies.

The implications of CRS and ICANS in CNS autoimmune populations require particular attention due to several disease-specific factors. Patients with MS, AE, or NMOSD may already have baseline cognitive dysfunction, compromised BBB integrity, or increased seizure susceptibility, potentially amplifying their consequences [42,43]. Furthermore, the pre-existing neuroinflammatory profile in these conditions may alter cytokine dynamics and CAR T-cell trafficking and activity patterns compared to systemic malignancies. For example, the presence of TLFs within the CNS in MS patients could create focal areas of localised inflammatory responses due to the localised high antigen density.

The long-term implications of prolonged B-cell aplasia and hypogammaglobulinemia raise important ethical and clinical considerations in patients with non-life-threatening chronic diseases, where the risk-benefit profile differs fundamentally from terminal malignancies. Unlike cancer patients facing imminent mortality, individuals with MS, AE, or NMOSD typically have years to decades of life expectancy, making the acceptable threshold for treatment-related morbidity considerably lower. The prospect of indefinite immunoglobulin replacement therapy, increased infection susceptibility, and potential long-term immune system alterations must be weighed against the chronic but generally non-fatal nature of these autoimmune conditions.

A separate safety consideration is on-target, off-tumor toxicity, which refers to recognition of the intended antigen on healthy tissues when expressed outside the target population. A notable example is a 2010 phase I trial in which a patient with metastatic colorectal cancer received third-generation anti-HER2/ERBB2 CAR T-cells and developed respiratory failure within minutes, dying hours later. The event was traced to recognition of physiologic HER2 on lung epithelium, triggering a massive cytokine surge [73]. This phenomenon has not been observed with anti-CD19 CAR Tcells. CD19 expression is highly restricted to the B-cell lineage, with no meaningful expression on other cells. Therefore, HER2-like fatal on-target, off-tumor injury to vital organs, including the brain, is considered highly unlikely on current evidence with anti-CD19 and similar anti-B-cell CAR T-cell therapies. The expected on-target, off-“tumor” effect of e.g., anti-CD19 CAR T-cell therapies is B-cell aplasia with hypogammaglobulinemia, reflecting the expected depletion of both pathogenic and non-pathogenic B-cells expressing CD19. This can result in immune compromise; immunoglobulin replacement therapy can be utilized if needed.

### 5.2. iCasp9 Suicide System [74]

As previously discussed, the use of CAR T-cell therapy can be accompanied by adverse side effects. To improve safety, researchers have developed a CAR T-cell “suicide” system designed to enable the elimination of infused CAR T-cells in the event of severe toxicity. This strategy involves the co-expression of an inducible caspase-9 (iCasp9) safety switch, which can trigger downstream apoptotic signalling upon administration of a chemical inducer of dimerization (CID).

Although no currently approved CAR T-cell products or clinical trials in CNS disease incorporate this system, preclinical studies in animal models investigating this approach have presented highly promising results, showing that iCasp9 can effectively deplete CAR T-cells following CID administration. CAR T-cells were monitored on days 7 and 14 post-infusion, demonstrating their persistence, and on day 14 CID was administered, leading to dimerization and activation of the iCasp9 proteins. Within three days (day 17), CAR T-cell signal intensity was almost eliminated, with even less signal measured by day 22 (eight days post-CID). In contrast, a control group receiving no CID showed stable CAR T-cell persistence over the same time. Development of such safety-switch technologies provides an important safeguard when severe adverse effects occur. However, despite encouraging preclinical data, the incorporation of inducible suicide switches such as iCasp9 into routine clinical CAR T-cell products is not anticipated in the immediate future and will require further validation of safety, reliability, and regulatory feasibility. Incorporating these approaches could significantly enhance the risk–benefit profile of CAR T-cell therapies and may, if supported by future clinical studies, expand their applicability to less severe forms of disease, including CNS autoimmunity.

## 6. The Plasticity in Different CAR T-Cell Constructs

Plasticity in CAR constructs involves several parameters. One key parameter is the antigen targeted. This flexibility allows us to explore disease-specific cellular targets to more precisely eliminate autoreactive populations and spare healthy subsets according to the pathophysiology behind different CNS autoimmune conditions. Another important parameter is the intracellular signalling domain which varies across CAR generations. This domain translates antigen-recognition into signalling and activation of the CAR T-cell. By modifying these signalling motifs, we can influence the activity and persistence of the therapy, tailoring the CAR T-cell therapy intensity accordingly. A newer concept currently in proof-of-concept development involves expressing the CAR on different cell lines. This experimental approach could allow us to harness other cellular capacities to become selectively activated, rather than remaining purely cytotoxic. Examples include the expression of CAR on immunosuppressive but not cytotoxic T-cells like Tregs though significant manufacturing complexity and safety validation barriers remain before clinical translation.

Different approaches to CAR expression are also under study. The current standard involves transducing T-cells with viral vectors carrying the CAR transgene, but other strategies, including mRNA-based transient CAR expression, are being explored. Finally, an area of interest is the source of the T-cells used to generate CAR T-cells and whether they are coming from the individual patient (autologous) or from a donor (allogeneic).

### 6.1. Allogeneic CAR T-Cells

**Figure 7:**
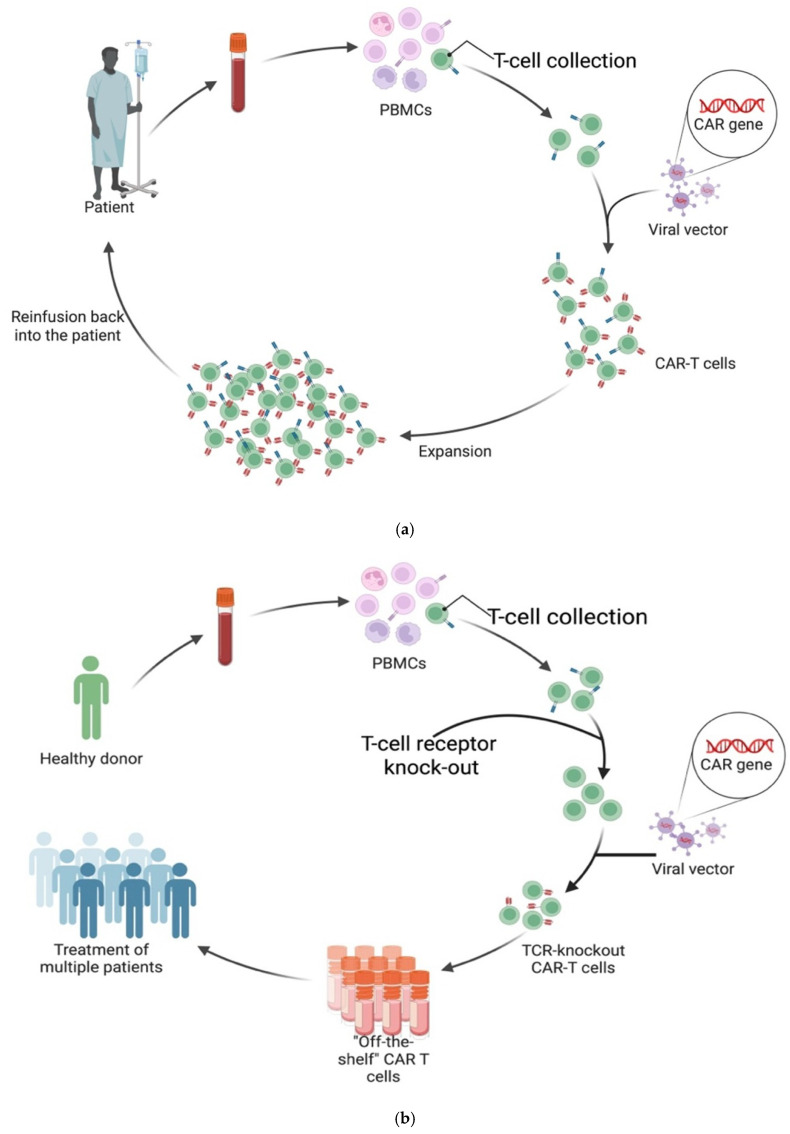
(**a**) Autologous chimeric antigen receptor (CAR) T-cell manufacturing workflow. Peripheral blood is collected from the patient to obtain peripheral blood mononuclear cells (PBMCs), from which T-cells are isolated. These T-cells are then genetically modified using a viral vector that introduces the CAR gene, creating CAR T-cells capable of recognising and targeting the cell that expresses an antigen for which the CAR has affinity and specificity. They are then expanded and infused back into the patient [76]. “Created in BioRender. Demetriou, F. (2025) https://BioRender.com/jw8pks9”. (Accessed date on 14 December 2025) (**b**) Production of allogeneic or “off-the-shelf” CAR T-cells. Peripheral blood is collected from healthy donors to obtain peripheral blood mononuclear cells (PBMCs), from which T-cells are isolated. Endogenous T-cell receptor (TCR) is knocked out to prevent graft-versus-host disease (GVHD) following infusion. The modified T-cells are then transduced with a viral vector carrying the CAR gene, generating TCR-knockout CAR T-cells. These engineered cells are expanded and cryopreserved as standardised “off-the-shelf” products that can be used to treat multiple patients [80]. “Created in BioRender. Demetriou, F. (2025) https://BioRender.com/jw8pks9”. (Accessed date on 14 December 2025).

CAR T-cell preparation typically begins with leukapheresis, a procedure that collects T-cells from the patient (in autologous therapy) or a donor (in allogeneic therapy). T-cells are often selected for surface markers indicative of differentiation state and functionality. Once collected, these T-cells are genetically modified to express the CAR, most commonly using viral vectors such as lentiviruses or retroviruses. These vectors deliver DNA encoding the CAR transgene, which integrates into the host T-cell genome, leading to stable CAR expression. Following successful engineering and expansion of the CAR T-cell population, they are reinfused back into the patient [75] (See Figure 7a).

All six currently FDA-approved CAR T-cell products are autologous. Allogeneic CAR T-cells have significant advantages, and ongoing clinical trials are exploring them. As the name suggests, allogeneic CAR T-cells are not derived from the individual patient but are an off-the-shelf option using T-cells collected from healthy donors (See Figure 7b). Significant advantages come with this, as the preparation of autologous CAR T-cells takes over two weeks and requires extensive specialised facilities, with costs ranging into the hundreds of thousands of dollars [76].

Additionally, autologous CAR T-cells fail to respond in 10–20% of patients who receive them in oncology, while autoimmunity data are early but growing [77]. Failure in response arises because harvesting T-cells from the individual patient carries risks regarding the quality and, especially, the quantity of leukocytes when patients undertake treatment protocols involving immunosuppressive and/or lymphodepleting agents [78]. Treatment-related T-cell lymphopenia excludes patients from receiving autologous CAR T-cell therapy or makes retreatment unfeasible. T-cell fitness also varies with factors such as age and chronic infections [79]. Unlike autologous products, allogeneic CAR T-cells are not limited by the patient’s own T-cell quantity or quality. Donor-derived T-cells tend to be more “fit” than patient cells and can be banked in preset dose ranges [80].

The main limitation in safely using allogeneic products is the potential of Graft-versus-Host Disease (GVHD) or Host-versus-Graft Disease (HVGD). GVHD involves the CAR T-cells recognising the host’s tissue as foreign and attacking it; this can result from incomplete knockout of the native TCRs during allogeneic preparation. Whereas HVGD involves the host recognising the CAR T-cells as foreign and eliminating them due to human leukocyte antigen (HLA) mismatch [81].

One allogeneic CAR T-cell example being investigated is the so-called azer-cel from TG Therapeutics, which was granted FDA approval for a Phase I clinical trial in 2024 for its application in autoimmune diseases and is currently recruiting patients (NCT06680037). Other allogeneic CAR T-cells under investigation are included in Table 2.

### 6.2. Anti-N-Methyl-D-Aspartate Receptor (NMDAR)-Specific Chimeric Autoantibody Receptor (CAAR) T-Cells for NMDAR Autoimmune Encephalitis (AE)

Autoimmune encephalitides (AEs) are characterised by autoantibody production against specific neuronal or glial antigens. Among these, NMDAR encephalitis is the most common subtype. The NMDAR is a glutamate-gated ion channel that plays a key role in synaptic transmission, synapse formation, and plasticity, thereby contributing to learning and memory [82].

**Figure 8:**
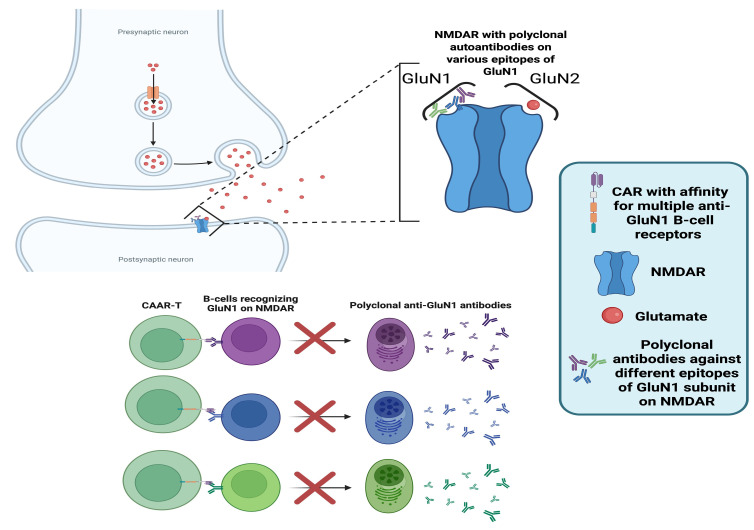
Mechanism of anti-N-methyl-D-aspartate receptor (NMDA)–specific chimeric autoantibody receptor (CAAR) T-cells in anti-NMDAR autoimmune encephalitis (AE). The left panel depicts the pathophysiology of anti-NMDAR AE, where polyclonal autoantibodies targeting the glutamate (N-methyl-D-aspartate) receptor subunit 1 (GluN1) of NMDA receptors disrupt synaptic signalling. The right panel illustrates the mechanism of CAAR T-cells engineered to express an NMDA receptor–derived extracellular domain that binds multiple B-cell receptors (BCRs) on anti-GluN1 B-cells. These CAAR T-cells eliminate pathogenic B-cells, preventing their differentiation into plasma cells. The ability of the same CAR to simultaneously recognise and bind multiple BCRs is depicted as the elimination of the polyclonal pool of plasma cells, which is the goal to achieve disease remission [87,88]. “Created in BioRender. Demetriou, F. (2025) https://BioRender.com/jw8pks9” (accessed on 14 December 2025).

The immunopathological mechanism of NMDAR AE involves polyclonal autoantibodies with affinity against the glutamate (N-methyl-D-aspartate) receptor subunit 1 (GluN1) of NMDAR (see Figure 8), resulting in NMDAR loss of function with subsequent synaptic changes that manifest as psychosis, epileptic episodes, and loss of cognitive function, with encephalopathy features [83,84,85,86]. NMDAR AE involves the presence of autoantibodies in both CSF and serum. The source of these autoantibodies are short-lived plasma-cells continuously repopulated by the differentiation of anti-NMDAR memory B-cells [87].

Due to the predominantly autoantibody-mediated pathology, a recent proof-of-concept study investigated NMDAR-specific chimeric autoantibody receptor (CAAR) T-cells in ex-vivo cultures of PBMCs from patients with neuronal autoimmune immunoglobulin-G–mediated autoimmune encephalitides, demonstrating selective targeting of NMDAR-specific autoreactive B-cells [88]. This CAR construct specifically recognises the B-cell receptor (BCR) of NMDAR-specific B-cells, to specifically target the autoantibody-producing populations while sparing healthy subsets. This was achieved by utilising an NMDAR autoantigen extracellular domain.

The repertoire of autoantibodies in NMDAR AE involves polyclonal IgG antibodies with affinity to various epitopes on the GluN1 subunit of NMDAR [87]. Therefore, the CAR construct was designed to simultaneously recognise variable BCRs, as the polyclonal antibody-pool must be eliminated for successful disease remission.

Polyclonality of autoantibodies is a major issue faced when designing autoantibody-specific CAAR T-cells, as the CAR needs to recognise a broad spectrum of pathogenic BCRs while avoiding recognition of healthy antigens, a requirement that significantly complicates receptor design and increases the risk of incomplete targeting or off-target toxicity.

The study did not involve humans but presented promising results in mouse models, both in vivo and in vitro. NMDAR-CAAR T-cells were successfully activated with human autoantibodies, secreted effector molecules, and killed targeted cells in vitro. Moreover, they proliferated upon encountering target cells and eliminated pathogenic populations in vivo without signs of therapy-induced toxicity.

This CAR construct highlights the plasticity of CAR design, a key strength of this revolutionary therapeutic platform. Currently, there are no approved therapies for AE, but there is an ongoing phase III trial (NCT05503264) investigating satralizumab in NMDAR and Leucine-rich glioma-inactivated 1 (LGI1) AE patients, along with inebilizumab (NCT04372615) and bortezomib (NCT03993262) in phase IIb clinical trials.

### 6.3. Anti-BCMA CAR T-Cells in NMOSD

**Figure 9:**
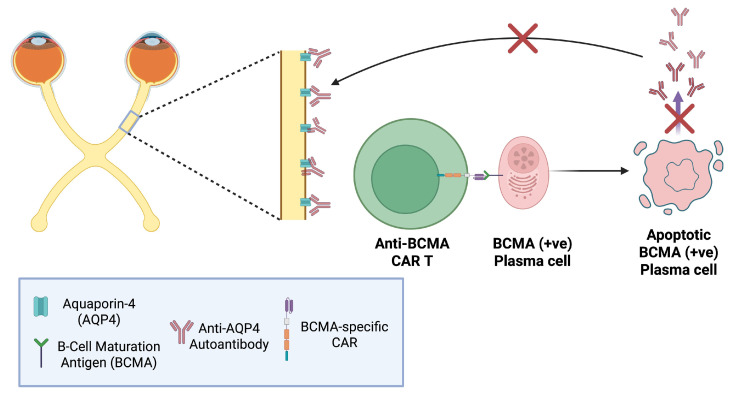
Anti-B-cell maturation antigen (BCMA) chimeric antigen receptor (CAR) T-cells in neuromyelitis optica spectrum disorders (NMOSD). Plasma cells produce pathogenic anti-aquaporin-4 (AQP4) autoantibodies that target AQP4 channels on astrocytes mainly within the optic nerve and spinal cord, leading to inflammation and demyelination. Anti-BCMA CAR T-cells are engineered to recognise BCMA expressed on late plasma cells and plasmablasts. Upon engagement with BCMA, the CAR T-cells get activated and induce apoptosis, eliminating the source of anti-AQP4 autoantibodies [90,92]. “Created in BioRender. Demetriou, F. (2025) https://BioRender.com/jw8pks9” (accessed on 14 December 2025).

NMOSD was long considered a variant of MS until the discovery of anti–aquaporin-4 (AQP4) antibodies in 2004 by Lennon et al. [89]. Polyclonal, plasma cell–derived IgGs primarily bind AQP4 on astrocytes of the optic nerve (see Figure 9), but also the spinal cord, and other specific CNS locations, triggering complement activation, inflammation, astrocyte loss, and demyelination.

First-line therapy has traditionally combined high-dose intravenous (IV) methylprednisolone for acute attacks with targeted mAb immunotherapies; however, 25–60% of NMOSD patients on these regimens still experience relapses and permanent disability [90]. B-cell–depleting mAbs reduce relapse rates but often fail to lower circulating anti–AQP4 titres, since long-lived plasma cells may lack CD19/CD20 expression [91].

Unlike MS, in which CD19/CD20 (+ve) B-cells, among others, act as APCs, sustaining T-cell–mediated demyelination, NMOSD is primarily an autoantibody-driven astrocytopathy. This distinction and the usual lack of CD19 and CD20 expression on plasma cells, support the rationale for anti-BCMA CAR T-cells instead of anti-CD19 or anti-CD20. BCMA is a TNF receptor family member upregulated on late stage plasmablasts and mature plasma cells [92]. BCMA-targeted CAR T-cell therapies aim to eliminate BCMA(+ve)/CD19/CD20(-ve) B-cells which anti-CD19/CD20 CAR T-cells cannot, while sparing non-antibody-secreting B-cells.

Recently introduced therapies like anti-C5, such as ravulizumab, have shown outstanding therapeutic outcomes, achieving up to 98.6% reduction in relapse risk [55]. Thus, investigation of CAR T-cells would be ethically justified only in cases where current DMTs including the newly introduced, fail.

### 6.4. MOG-Specific CAR T-Regulatory Cells (CAR Tregs) in MS

CAR Tregs represent an alternative approach to treat multiple sclerosis (MS) compared to anti-CD19 CAR T-cells. As previously discussed, CD19 is expressed in most B-cell populations; thus, anti-CD19 CAR T-cells can induce deep B-cell depletion, potentially leading to opportunistic infections due to extensive immunosuppression until protective immune populations reconstitute.

**Figure 10:**
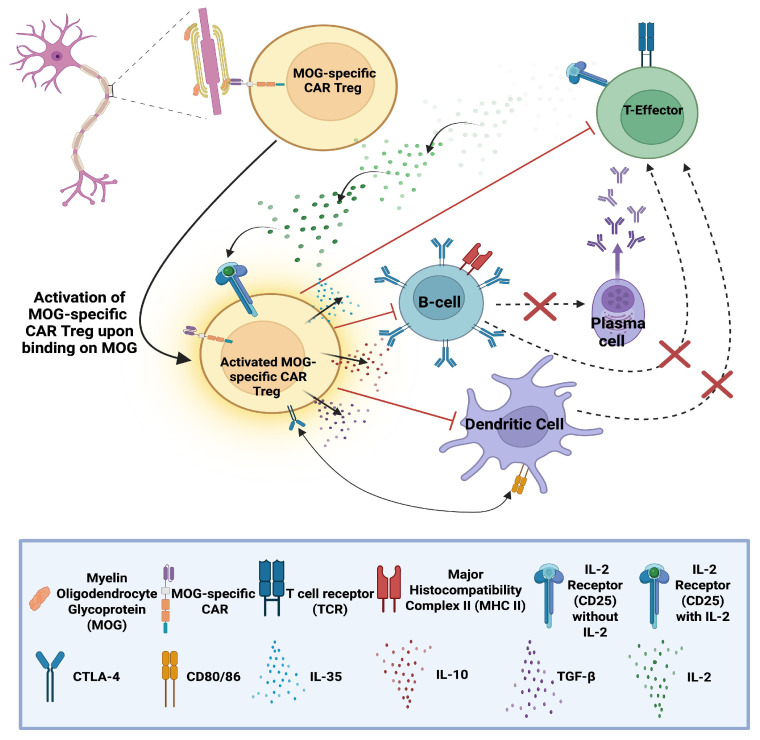
Myelin oligodendrocyte (MOG)-specific chimeric antibody receptor (CAR) Tregs recognise and bind MOG, leading to their activation. Upon activation, they secrete immunosuppressive cytokines (IL-10, IL-35, and TGF-β) suppressing among others B-cell activation and differentiation into antibody-producing plasma cells and upregulate CTLA-4 which interacts with its ligands (CD80 & CD86) on antigen-presenting-cells (APCs) limiting T-effector activation. Expression of CD25 consumes IL-2, depriving effector T-cells of this growth factor, inhibiting them [93,94]. “Created in BioRender. Demetriou, F. (2025) https://BioRender.com/jw8pks9” (accessed on 14 December 2025).

The experimental capacity to engineer CARs on different cell types has enabled the exploration of anti-MOG CAR Tregs as an alternative to conventional cytotoxic anti-CD19 CAR T-cells in preclinical models [93]. In contrast to cytotoxic CAR T-cells that recognise and eliminate e.g., CD19 (+ve) B-cells, MOG-specific CAR Tregs aim to harness the immunosuppressive capacities of regulatory T-cells to silence the immune response against myelin oligodendrocyte glycoprotein (MOG) without directly killing B-cells.The immunosuppressive mechanisms of MOG-specific CAR Tregs involve CAR-mediated recognition of native MOG in the CNS, which activates Tregs and promotes the release of anti-inflammatory cytokines, such as IL-10 and TGF-β. These molecules inhibit locally resident immune populations and are expected to upregulate immune checkpoint receptors, such as cytotoxic T-lymphocyte–associated protein 4 (CTLA-4) and programmed cell-death-protein 1 (PD-1) (see Figure 10) [94].

A preclinical study [93] investigated the capacity of human MOG-specific CAR Tregs in inhibiting the proliferation of CD4 (+ve) conventional T-cells (Tconvs) ex vivo. The MOG-specific CAR Tregs demonstrated antigen-specific activity, significantly inhibiting Tconv proliferation only upon MOG stimulation, following pre-stimulation with MOG-coated plates or anti-CD3/CD28 beads (control).

This antigen-specific regulatory approach represents a promising direction for precision immune modulation in MS, offering potentially therapeutic effects without the broad immunosuppression observed with cytotoxic CAR T-cell therapies.

Despite these promising findings, a key limitation of CAR Treg–based therapies remains the challenge of ensuring Treg stability and sustained suppressive function in vivo. Regulatory T-cells exhibit a degree of phenotypic plasticity, and exposure to the inflammatory cytokine profiles present in active MS may compromise their lineage stability. In particular, loss of FOXP3 expression or conversion into effector-like cells could inhibit their immunosuppressive capacity or, in the worst case, exacerbate pathogenic immune responses. Additional challenges include maintaining long-term CAR Treg persistence and preventing exhaustion within a chronic inflammatory environment. These factors collectively complicate clinical translation and underscore that, while the concept sounds very promising, extensive research is still required to transform this promising approach into clinical reality for patients with CNS autoimmune conditions.

### 6.5. Peptide-Specific CAR T-Cells (pMHCII CAR T-Cells) for the Selective Depletion of Autoreactive T-Cells [95]

**Figure 11:**
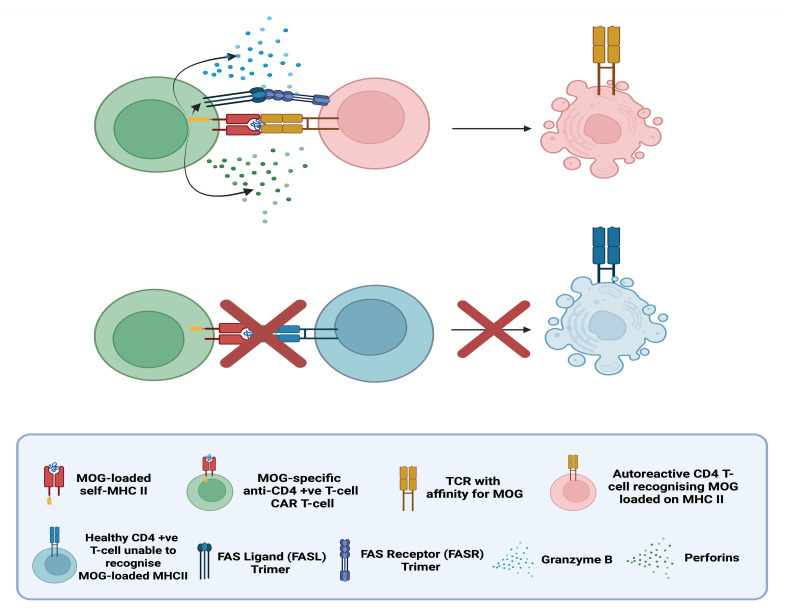
Schematic illustration of the selective elimination of autoreactive CD4 (+ve) T-cells recognising myelin oligodendrocyte glycoprotein (MOG) by peptide (MOG)-specific chimeric antigen receptor (CAR) T-cells (MOG-MHCII-CAR T-cells). Top: A pMHCII-CAR T-cell engineered to express an MHC II molecule loaded with myelin oligodendrocyte glycoprotein (MOG) selectively engages autoreactive CD4 (+ve) T-cells whose T-cell receptors (TCRs) recognise MOG presented by antigen-presenting cells (APCs). This antigen-specific interaction triggers targeted elimination of the autoreactive CD4 (+ve) T-cell. Bottom: CD4^+^ T-cells that do not recognize MOG do not bind the MOG–MHCII CAR and are spared, demonstrating the specificity of this CAR design for autoreactive CD4 (+ve) T-cells [95]. “Created in BioRender. Demetriou, F. (2025) https://BioRender.com/jw8pks9” (accessed on 14 December 2025).

In 2022 in a preclinical study in mice with experimental autoimmune encephalomyelitis (EAE) [95], introduced the concept of replacing the traditional CAR recognition domain (an antibody-derived scFv), with an MHC II molecule loaded with self-peptides (see Figure 11). MHC II is expressed by APCs presenting to and activating CD4 (+ve) T-helper cells which in-turn promote inflammation and activate other immune effectors including cytotoxic CD8 (+ve) T-cells. By using MOG-loaded MHC II molecules as the extracellular domain, the peptide-specific CAR can selectively bind self-reactive T-cells whose TCRs recognise MOG presented by APCs. Upon encountering a CD4 (+ve) T-cell expressing a MOG-specific TCR, the MOG-MHCII-CAR T-cell gets activated and eliminates the autoreactive CD4 (+ve) T-cell.

They demonstrated that these pMHCII-CAR T-cells can efficiently eliminate both naïve and activated antigen-specific CD4 (+ve) T-cells in vivo. Importantly, the sensitivity of the CAR was influenced by TCR affinities: the original lower-sensitivity MOG35–55 pMHCII-CAR preferentially eliminated high-affinity MOG-specific T-cells, including Treg clones, whereas many lower-affinity effector T-cells persisted. Engineering improvements that enhanced CAR stability and signalling strength, increased sensitivity, enabling broader deletion of both high- and low-affinity autoreactive T-cells. These findings highlight the critical role of TCR affinity in autoimmune pathogenesis and suggest that pMHCII-CAR designs could be explored to exclusively target autoreactive T-cells.

A significant challenge for this approach is avoiding fratricide, which is the unwanted elimination of the therapeutic CAR T-cells themselves. Since the CAR’s extracellular domain is a self pMHCII complex, there is a risk that the engineered cells could recognize and kill each other if they express a TCR specific for the same self-peptide. Successful clinical translation will therefore require careful strategies, such as selecting CAR T-cell donors with non-reactive TCRs or genetically editing the therapeutic cells to remove their endogenous TCR, ensuring the therapy targets only T-cells without self-destructing. To date, no human trials active or planned investigate this concept, but it still shows potential future capacities this therapy might have to offer.

### 6.6. mRNA-Induced CAR T-Cells

Conventional CAR T-cell manufacturing involves transducing patient-derived T-cells with viral vectors, leading to permanent CAR expression in each engineered and descendant cell. While this approach yields potent, long-lived CAR T-cells, it also results in unpredictable PK/PD profiles [96]. This unpredictability is among the reasons CAR T-cell therapies are confined to last-resort use in refractory or aggressive cases. Additionally, with d-transduced CARs, lymphodepletion therapy, a process used to create optimal conditions for successful in vivo expansion, is not required. The risks associated with lymphodepletion include cytopenia and an added risk of opportunistic infections due to significantly reduced lymphocyte counts.

In contrast, mRNA-based CAR T-Cell therapies offer predictable persistence and a level of intensity that can be easily manipulated. mRNA has a defined half-life, which reflects the duration of CAR expression. The mRNA molecule can be delivered to T-cells to create CAR T-cells using electroporation which results in CAR expression for roughly a week [97,98]. This transient expression results in a more controllable PK/PD profile, minimising the risk of prolonged or excessive activity, potentially constituting mRNA CAR T-Cell therapies a choice for less aggressive forms of diseases.

However, this finite persistence while advantageous for predictability and safety, it also represents a fundamental limitation of mRNA-induced CAR T-cell therapies. The transient nature of CAR expression precludes durable in vivo expansion and typically necessitates repeated dosing to maintain therapeutic efficacy, thereby increasing treatment frequency and patient burden. In this regard, mRNA CAR T-cell therapies may resemble current DMTs, which require ongoing or cyclical administration to sustain disease control. Consequently, while mRNA-based CAR T-cells offer an attractive safety and controllability profile, their clinical positioning may be best suited to scenarios where reversible immune modulation is desired, rather than as a definitive immune-reconstituting strategy. By contrast, permanently engineered CAR T-cell products continue to hold conceptual appeal in settings where the goal is long-lasting disease remission achieved with fewer or potentially single-treatment courses, provided safety and tolerability can be adequately managed.

A clinical trial currently in phase 1b/2a released data in 2023 on the safety and efficacy of an autologous RNA CAR T-Cell (rCAR T), called Descartes-08, in patients with Myasthenia Gravis (MG) [46]. Fourteen patients were enrolled, and follow-ups with a median of 5 months showed no dose-limiting toxicities, CRS, or ICANS. Descartes-08 administration resulted in decreases in MG severity that persisted at the most recent follow-up at 9 months.

## 7. Active or Planned CAR T-Cell Clinical Trials Which Include CNS Autoimmunity Patients

Current CAR T-cell clinical trials in CNS autoimmunity (See Table 2) are uniformly restricted to patients with relapsing and/or refractory disease after failure of standard or high-efficacy treatments. The majority of studies employ CD19- or CD20-directed CAR T-cells, aiming for broad B-cell depletion (as these CDs are the most broadly expressed); however, an increasing number of trials are exploring BCMA-targeted strategies, which selectively eliminate plasma-cells and directly address the autoantibody-driven component of autoimmunity. Importantly, some plasma cells lack CD19 allowing them to evade anti-CD19 therapies [91]. These distinctions may be particularly relevant for predominantly autoantibody-mediated disorders such as NMOSD, MOGAD and AE.

Allogeneic CAR T-cells explored present with significant theoretical capacities, as over autologous approaches they can come with improved scalability, reduced manufacturing costs, and more predictable activity. In contrast, patient-derived CAR T-cells may vary substantially in their quality and functional capacity in different batches collected from each patient [80,99]. At the same time, bispecific [100] and trispecific [101] CAR T-cell constructs are investigated to achieve more profound and durable elimination of B-cells by increasing the targeted antigens and combat the potential of antigen loss. Importantly, this broader targeting strategy, may also increase the risk of off-target effects and immune-related toxicity. Finally, the extended estimated completion timelines of many ongoing and planned trials, often spanning several years, underscore both the early developmental stage of CAR T-cell therapy in CNS autoimmunity and the significant safety, and regulatory challenges that must be addressed before broader clinical application can be considered.

**Table 2:** CAR T-cell clinical trials that include CNS autoimmune diseases.

	Condition	NCT no.	Antigen Target	Phase	Patient enrolment	Study Start	Study Completion
1	Relapsed/Refractory NMOSD	NCT03605238	CD19/CD20	I * Withdrawn due to recruitment issues	N/A	N/A	N/A
2	(AQP4 +ve) NMOSD Recurrent & Refractory	NCT05828212	CD19	I	9 (actual)	05-2023	30-7-2025 (actual)
3	Refractory Primary/ Secondary Progressive MS	NCT06384976	CD19	II	120 (target)	09-2024	01-2029 (estimated)
4	Relapsing/Progressive MS Refractory MG	NCT06220201	CD19	I	120 (estimated)	03-2024	07-2027 (estimated)
5	Non-relapsing/Progressive MS	NCT06138132	CD19	I	12 (estimated)	04-2024	2027-06 (estimated)
6	Refractory MS/not responding to standard therapy	NCT06451159	CD19	I	10 (estimated)	06-2024	2027-06 (estimated)
7 *	Relapsing/Refractory NMOSD, MG, MS, MOGAD, CIDP, IIM, AE, POEMS syndrome	NCT04561557	(Allogeneic) BCMA	I	36 (estimated)	09-2020	05-2031 (estimated)
8 **	Refractory to Standard Therapy SLE, IMNM, NMOSD, MS, MG, SSc	NCT06249438	Bispecific CD20&BCMA	I	30 (estimated)	03-2024	03-2040 (estimated)
9 *	Refractory, failed standard treatment or lack effective treatment NMOSD, gMG, CIDP, MS	NCT06485232	Allogeneic CD19 & BCMA	I	25 (estimated)	02-2025	12-2027 (estimated)
10 ***	NMOSD, MOGAD, MS, MG	NCT06869278	Trispecific CD19 & CD20 & CD22	I	37 (estimated)	06-2025 (estimated)	12-2029 (estimated)
11	MS, NMOSD, IIT, SLE, SSc, AAV, IIM, Sjögren’s Syndrome (SS), MG	NCT06548620	CD19	I	18 (estimated)	08-2024	08-2027 (estimated)
12	Refractory Neuroimmune Diseases	NCT07022197	BAFFR	I and II	27 (estimated)	04-2025	12-2027 (estimated)
13	Relapsing Multiple Sclerosis (MS)	NCT06617793	CD19	I and II	28 (estimated)	02-2025	10-2030 (estimated)
14	Non-active Progressive Multiple Sclerosis (PMS)	NCT06675864	CD19	I and II	28 (estimated)	12-2024	06-2030 (estimated)
15	Relapsing/Progressive MS	NCT07006805	CD19	I and II	12 (estimated)	06-2026	10-2029 (estimated)
16 *	Relapse/Refractory Neurological Autoimmune Diseases	NCT06939166	CD19/ BCMA Allogeneic	I	12 (estimated)	06-2025 (estimated)	10-2027 (estimated)
17 *	B-cell Mediated Autoimmune Disorders	NCT06680037	CD19 Allogeneic	I	32 (estimated)	2025-05-06 (actual)	2029-01-01 (estimated)

* Allogeneic; ** Bispecific; *** Trispecific; Central Nervous System (CNS); Neuromyelitis Optica Spectrum Disorders (NMOSD); Multiple sclerosis (MS); Myasthenia Gravis (MG); autoimmune encephalitis (AE); Myelin Oligodendrocyte Glycoprotein Antibody-Associated Disease (MOGAD); Chronic Inflammatory Demyelinating Polyradiculoneuropathy (CIDP); Sjögren’s Syndrome (SS); ANCA associated vasculitis (AAV); Systemic Sclerosis (SSc); Idiopathic Inflammatory Myopathy (IIM).

## 8. Case Reports of CAR T-Cell Use in CNS Autoimmunity

The available case reports on the use of CAR T-cells in CNS autoimmunity are encouraging but too limited to allow firm conclusions. To date, 15 patients have been reported: 2 with MS, 1 with MOGAD, and 12 with NMOSD. Results from ongoing and planned studies will be crucial for validating these preliminary observations and determining the safety and efficacy of CAR T-cell therapy in CNS autoimmune diseases.

### 8.1. Anti-CD19 CAR T-Cells in 2 MS Patients [102]

Early clinical experience with CD19-directed CAR T-cell therapy in MS is currently limited to two case reports. Kyverna Therapeutics reported the use of KYV-101, a second-generation anti-CD19 CAR T-cell, in two patients with advanced MS, one with long-standing RRMS and one with PPMS. Both patients received lymphodepleting chemotherapy followed by a single CAR T-cell infusion.

First Patient: A 47-year-old woman with a 23-year history of RRMS and progressive gait impairment despite prior anti-CD20 therapy (ocrelizumab) received CD19-directed CAR T-cell therapy following lymphodepletion. Baseline imaging demonstrated extensive cerebral and spinal disease burden (>50 lesions). Post-infusion, the patient developed low-grade CRS, manifested by recurrent fever and transient facial and neck swelling, which was successfully managed with tocilizumab and corticosteroids. No ICANS was observed. A transient worsening of pre-existing spastic paresis occurred and was interpreted as Uhthoff’s phenomenon, with temporary EDSS worsening that returned to baseline by day 29. Mild, self-limited transaminase elevation was noted. Follow-up to day 100 revealed no ongoing treatment-related toxicity. MRI at day 64 showed a single new, non-enhancing thoracic spinal cord T2 lesion that remained stable at day 100. Functionally, self-reported walking distance improved from 400 m at baseline to 700 m at day 100, while other standardized neurological assessments remained unchanged.

Second patient: A 36-year-old man with PPMS and severe gait impairment despite prior ocrelizumab therapy received CD19-directed CAR T-cell therapy after lymphodepletion. At baseline, the patient required a walker (maximum walking-distance ~10 m), and MRI demonstrated more than 20 disseminated CNS lesions. Throughout follow-up to day 28 post-infusion, no CRS or ICANS was observed. MRI at day 14 remained unchanged. A transient elevation in liver transaminases occurred and improved with ursodeoxycholic acid treatment. No additional adverse events or new neurological symptoms were reported, and disability remained stable over the observation period. Significant lymphocyte suppression was observed, comparable to the findings in patient 1.

The extremely small sample size precludes any statistically significant conclusions. Additionally, the short duration of follow-up is insufficient to assess long-term safety, efficacy, or the durability of B-cell depletion. The open-label, uncontrolled nature of these case reports means observed changes could be influenced by the natural fluctuation of MS, patient expectation, or supportive care, rather than a direct therapeutic effect. Furthermore, both patients had received prior potent B-cell depletion with ocrelizumab, making it difficult to isolate the specific impact of CAR T-cells from the effects of previous therapy. Therefore, while these initial reports provide essential proof of feasibility and short-term tolerability, they should not be overinterpreted as evidence of a cure or definitive clinical efficacy; they underscore the need for larger, controlled trials with prolonged observation.

### 8.2. Anti-CD19 CAR T-Cells in a 25-Year-Old Male Patient with Refractory Myelin Oligodendrocyte Antibody-Associated Disease (MOGAD)

MOGAD is another autoimmune CNS disease characterised by demyelination. MOGAD in the past was also under the umbrella of MS. MOG-specific antibodies are found in the CSF and serum of 85% of patients, and OCBs in 15% [103] of patients with MOGAD.

A case report [104] describes the use of anti-CD19 CAR T-cells in a patient who, at the age of 18, presented with sensory disturbances in his lower limbs, later accompanied by paresis and impaired bladder emptying. Upon evaluation, MRI revealed a spinal cord lesion extending from T4 to the conus medullaris, with no lesions in the brain. Cell-based assays detected anti-MOG IgG only in the serum (titre 1:160), with no antibodies in the CSF, and AQP4-IgG was negative. Electrophoresis showed no OCBs. In the following years, the patient experienced two episodes of myelitis, followed by six episodes of optic neuritis (ON); ON is the most common initial symptom in MOGAD. Five months after his initial presentation, he developed relapsing sensory impairment, a new spinal cord lesion at T6, and persistent serum MOG-IgG positivity, which led to the initiation of long-term treatment with Rituximab.

All six episodes of ON were treated with intravenous methylprednisolone (IVMTP) and prednisone. In two of these episodes, plasma exchange was performed in conjunction with IVMTP. After the sixth ON episode, the patient underwent optic coherence tomography (OCT), which showed reduced retinal and inner plexiform layer thickness and decreased visual acuity. The aggressiveness of the disease made the patient eligible for autologous anti-CD19 CAR T-cell therapy (ARI-0001). The results of this CAR T-cell application are highly promising, raising hopes that it may become a therapy capable of curing MOGAD.

Upon the administration of CAR T-cells, complete elimination of CD-19 (+ve) B-cells was achieved by day +7, and the populations re-established in +141 days. The newly formed populations consisted of naïve B-cells and diminished or absent memory B-cells. At day +29, the patient experienced an episode of left ON even though he was already seronegative for MOG IgG. Since then, a year later, he remains healthy with no relapses and free of anti-MOG IgG. 

The fact that this is a case report involves a single patient and lacks controls, prevents us from drawing any significant conclusions. Additionally, right before the introduction of the CARs, patients undergo lymphodepleting therapy with cyclophosphamide. We cannot exclude the possibility that the observed results are not due to lymphodepletion from these therapies. This study provides class IV evidence as controls were not included.

### 8.3. Anti-BCMA CAR T-Cells in Twelve NMOSD Patients [72]

The largest clinical experience to date with CAR T-cell therapy in CNS autoimmunity involves a single-arm study of anti-BCMA CAR T-cells (CT103A) in 12 patients with relapsed or refractory AQP4-IgG–seropositive NMOSD. All patients had highly active disease despite prolonged treatment with corticosteroids and multiple immunosuppressive agents, with frequent relapses in the year preceding enrollment. CAR T-cell therapy was administered following lymphodepleting chemotherapy, and all background immunosuppressive therapies were subsequently discontinued.

From a safety perspective, all patients experienced grade ≥ 3 adverse events, predominantly hematologic toxicities related to conditioning chemotherapy, including leukopenia and neutropenia (100%), anemia (50%), and thrombocytopenia (25%), most of which resolved within four weeks. Infections occurred in 58% of patients, including cytomegalovirus reactivation, pneumonia, and coagulation disorders, underscoring the clinically relevant immunosuppressive burden CAR T-cell therapies. All patients developed low-grade (grade 1–2) cytokine release syndrome, but no patient developed ICANS. Serum AQP4-IgG titres declined in all patients, becoming negative in 70% by 12 weeks, while serum BCMA levels fell below detection within 1 month before returning to baseline by six months. CAR T-cells remained detectable in the peripheral blood for up to six months, with detection rates of 100% in the first month, 73% in two months, 60% in three months, and 17% in months.

Clinically, during a median follow-up of 5.5 months (range 1–14 months), 11/12 of patients achieved drug-free remission without reported relapses, accompanied by improvements in disability measures including EDSS, visual acuity, ambulation, and bowel and bladder function. However, interpretation of these outcomes requires caution. The study lacks a control group, follow-up duration remains short for a relapsing disease such as NMOSD, and all patients received lymphodepleting chemotherapy prior to CAR T-cell infusion, which itself may contribute to transient disease suppression. Additionally, one patient experienced a possible clinical relapse at 14 months, and the durability of both serologic remission and functional improvement remains uncertain.

Collectively, this study provides compelling proof-of-concept evidence that BCMA-directed CAR T-cells can induce profound biological and short-term clinical effects in severe, treatment-refractory NMOSD. However, the small sample size of twelve (12) patietns, absence of controls, heterogeneous follow-up, and significant treatment-related toxicity preclude conclusions regarding long-term efficacy, safety, or curative potential. Accordingly, these observations are preliminary and require confirmation in well-designed, controlled trials with extended follow-up prior to consideration for wider clinical use.

## 9. Discussion-Conclusions

The therapeutic landscape of CNS autoimmunity has evolved substantially over the past two decades, with DMTs achieving effective control of relapsing inflammatory activity in most patients. Within this context, CAR T-cell target a narrow and well-defined set of unmet needs that remain insufficiently addressed by existing approaches.

One such unmet need is the persistence of compartmentalised CNS immune pathology, particularly the presence of CNS-resident B-cell populations and TLFs. The limited CNS biodistribution of anti-B-cell mAbs, combined with their dependence on peripheral immune effector cells, constrains their ability to eliminate these protected immune niches. CAR T-cells, by contrast, are self-sufficient cytotoxic effectors and, as activated lymphocytes, may actively traverse the BBB, raising the possibility of direct targeting of CNS-resident pathogenic immune cells. This theoretical advantage provides a strong biological rationale for their investigation in CNS autoimmunity. At the same time other small-molecule therapies, including BTKIs are being investigated for their capacity to penetrate the CNS, from which CAR T-cells must show superiority in efficacy and risk-benefit balance to justify their use.

Broader clinical reality of CAR T-cell therapy remains restrictive. High costs, complex manufacturing and logistics, limited clinical experience in CNS autoimmune diseases, and the risk of severe adverse events including CRS and ICANS, currently confine their realistic application to patients with very severe, refractory disease in whom established therapies have failed. Importantly, in conditions where effective and comparatively safe DMTs are available, the exploration of CAR T-cell therapy outside such refractory settings would be difficult to justify ethically, given the current uncertainty surrounding their risk–benefit profile in CNS autoimmunity.

Another major theoretical appeal of CAR T-cell therapy lies in its potential to reduce long-term treatment burden. Chronic administration of immunomodulatory or immunosuppressive agents is associated with cumulative toxicity and long-term safety liabilities. Immune reconstitution strategies such as alemtuzumab and cladribine partially address this issue by enabling prolonged treatment-free intervals, yet their effects on CNS-resident immune compartments are indirect and incomplete. CAR T-cells offer the possibility of antigen-defined immune depletion and, potentially, a deeper and more durable form of immune reprogramming. Evidence from peripheral autoimmune diseases, such as in SLE, suggests that CD19-directed CAR T-cells can induce long-lasting, drug-free remission accompanied by qualitative changes in immune reconstitution. However, whether similar immune “resetting” can be reliably achieved in CNS autoimmunity remains unproven, particularly given the confounding effects of lymphodepleting conditioning regimens used in all reported cases to date and their small number.

In this early phase of clinical translation, B-cell–targeted CAR T-cell approaches represent the most rational initial strategy. Targeting CD19 and BCMA benefits from extensive clinical experience in oncology, established manufacturing platforms, and well-characterised safety profiles, providing the most robust translational framework for application in autoimmunity. Accordingly, these antigens also constitute the principal targets currently under investigation in clinical trials of CAR T-cell therapy for CNS autoimmune diseases. CD19-directed approaches are biologically aligned with the known roles of B-cells in the pathophysiology of MS, NMOSD, MOGAD, and autoimmune encephalitis, whereas BCMA-targeted CAR T-cells may be particularly relevant for predominantly autoantibody-mediated diseases through selective plasma-cell depletion. In contrast, more experimental platforms including CAAR T-cells, CAR Tregs, and pMHCII-CAR T-cells, highlight the modularity and disease-specific precision of CAR design but remain at a very early stage and cannot currently be considered clinically realistic options in the near future.

For CAR T-cell therapies to move beyond exceptional, highly refractory cases, several stringent conditions must be met. These include demonstration of an acceptable and predictable safety profile in larger cohorts, clear identification of patient subgroups most likely to benefit, and evidence of durable clinical efficacy exceeding that achievable with existing DMTs. In addition, feasibility and cost considerations will be decisive. Advances such as allogeneic “off-the-shelf” CAR T-cells and transient mRNA-induced CAR expression systems may prove pivotal by offering more predictable pharmacokinetic and pharmacodynamic profiles, reduced toxicity risk, improved scalability, and lower costs. Such developments could ultimately enable exploration of CAR-based therapies in less severe disease phenotypes, provided safety can be substantially improved.

In conclusion, CAR T-cell therapies hold the potential to reshape immune intervention in CNS autoimmunity, not as a replacement for current DMTs, but as a highly targeted strategy addressing specific unmet needs, particularly compartmentalised CNS immune pathology and the desire for durable disease control with reduced treatment burden. At present, their role remains investigational and confined to carefully selected patients with severe, treatment-refractory disease. Ongoing and future controlled clinical trials will determine whether advances in safety, targeting, and manufacturing can expand their applicability, or whether CAR T-cell therapy will remain a niche, but powerful option reserved for the most challenging clinical scenarios.

## Data Availability

No new data were created or analysed in this study.

## Abbreviations

The following abbreviations are used in this manuscript:

CNS	Central nervous system
DMT	Disease modifying therapy
BCMA	B-cell maturation antigen
CD	Cluster of differentiation
MS	Multiple sclerosis
AAV	ANCA associated vasculitis
IIM	Idiopathic inflammatory myopathy
SS	Systemic sclerosis
SSc	Sjögren’s syndrome
CIDP	Chronic inflammatory demyelinating polyradiculoneuropathy
mAbs	Monoclonal antibodies
NMOSD	Neuromyelitis optica spectrum disorder
MOGAD	Myelin oligodendrocyte antibody-associated disease
PML	Progressive multifocal leukoencephalopathy
AE	Autoimmune encephalitis
AEm	Autoimmune encephalomyelitis
CAR	Chimeric antigen receptor
MHC	Major histocompatibility complex
TCR	T-cell receptor
IL	Interleukin
DMTs	Disease modifying therapies
NFAT	Nuclear factor of activated T-cells
TNF	Tumour necrosis factor
BTKIs	Bruton’s tyrosine kinase inhibitors
TIL	Tumour infiltrating lymphocyte
CM	Co-stimulatory molecule
PSMA	Prostate-specific membrane antigen
TRUCKS	T-cells redirected for universal cytokine-mediated killing
ADCC	Antibody-dependent cellular cytotoxicity
ADCP	Antibody-dependent cellular phagocytosis
CDC	Complement-depended cytotoxicity
NK Cell	Natural killer cell
Neu	Neutrophil
MΦ	Macrophage
MAC	Membrane-attack complex
CSF	Cerebrospinal fluid
OCBs	Oligoclonal bands
SPMS	Secondary progressive MS
BBB	Blood-brain-barrier
TLF	Tertiary lymphoid follicle
VCAM-1	Vascular cell adhesion molecule-1
ICAM-1	Intercellular adhesion molecule-1
aHSCT	Autologous hematopoietic stem-cell transplantation
SLE	Systemic lupous erythematosus
CRS	Cytokine release syndrome
ICANS	Immune effector cell-associated neurotoxicity syndrome
CID	Chemical Inducer of dimerization
Treg	T-regulatory cell
CTLA-4	Cytotoxic T-lymphocyte–associated protein 4
PD-1	Programmed cell-death-protein 1
GVHD	Graft-versus-host disease
HVGD	Host-versus-graft disease
HLA	Human leukocyte antigen
PBMCs	Peripheral blood mononuclear cells
NMDAR	N-methyl-D-aspartate receptor
CAAR	Chimeric autoantibody receptor
BCR	B-cell receptor
LGI1	Leucine-rich glioma-inactivated 1
AQP4	Aquaporin-4
MOG	Myelin oligodendrocyte glycoprotein
TGF	Transforming growth factor
APCs	Antigen-presenting cells
PK	Pharmacokinetics
PD	Pharmacodynamics
MG	Myasthenia gravis
rCAR T	RNA CAR T-cell
RRMS	Relapse-refractory multiple sclerosis
PPMS	Primary-progressive multiple sclerosis
ON	Optic neuritis
IVMTP	Intravenous methylprednisolone
OCT	Optic coherence tomography
S1P	Sphingosine-1-Phosp
EMA	European Medicines Agency
EDSS	Expanded disability status scale
